# Epothilone B prevents lipopolysaccharide-induced inflammatory osteolysis through suppressing osteoclastogenesis via STAT3 signaling pathway

**DOI:** 10.18632/aging.103337

**Published:** 2020-06-11

**Authors:** Yueqi Chen, Yiran Wang, Junxian Hu, Yong Tang, Zhansong Tian, Wenhui Hu, Fanchun Zeng, Jiulin Tan, Qijie Dai, Zhiyong Hou, Fei Luo, Jianzhong Xu, Shiwu Dong

**Affiliations:** 1 Department of Biomedical Materials Science, Third Military Medical University (Army Medical University), Chongqing 400038, China; 2 Department of Orthopedics, Southwest Hospital, Third Military Medical University (Army Medical University), Chongqing 400038, China; 3 Department of Orthopaedic Surgery, Third Hospital of Hebei Medical University, Shijiazhuang 050051, Hebei, China; 4 The Key Laboratory of Trauma, Burns and Combined Injury, Third Military Medical University (Army Medical University), Chongqing 400038, China

**Keywords:** Epothilone B (Epo B), lipopolysaccharide (LPS), inflammatory osteolysis, osteoclastogenesis, STAT3

## Abstract

Inflammatory osteolysis is a common osteolytic specificity that occurs during infectious orthopaedic surgery and is characterized by an imbalance in bone homeostasis due to excessive osteoclast bone resorption activity. Epothilone B (Epo B) induced α-tubulin polymerization and enhanced microtubule stability, which also played an essential role in anti-inflammatory effect on the regulation of many diseases. However, its effects on skeletal system have rarely been investigated. Our study demonstrated that Epo B inhibited osteoclastogenesis *in vitro* and prevented inflammatory osteolysis *in vivo.* Further analysis showed that Epo B also markedly induced mature osteoclasts apoptosis during osteoclastogenesis. Mechanistically, Epo B directly suppressed osteoclastogenesis by the inhibitory regulation of the phosphorylation and activation of PI3K/Akt/STAT3 signaling directly, and the suppressive regulation of the CD9/gp130/STAT3 signaling pathway indirectly. The negative regulatory effect on STAT3 signaling further restrained the translocation of NF-κB p65 and NFATc1 from the cytosol to the nuclei during RANKL stimulation. Additionally, the expression of osteoclast specific genes was also significantly attenuated during osteoclast fusion and differentiation. Taken together, these findings illustrated that Epo B protected against LPS-induced bone destruction through inhibiting osteoclastogenesis via regulating the STAT3 dependent signaling pathway.

## INTRODUCTION

Decreased bone mineral metabolic activity was an essential aging associated characteristic. Along with increased age, the incidence of bone related infectious diseases has gradually increased. Infectious bone diseases including osteomyelitis, orthopaedic implant-associated infection and other severe inflammatory osteolysis destroyed dynamic bone matrix homeostasis (regulated by osteoclast-mediated bone resorption and osteoblast-mediated bone formation) [1–3]. In many clinical bone infection trials, efficacious means of treating these diseases were limited and mainly included surgical debridement, antibiotics, and nonsurgical mechanical treatment [4]. As a result, developing an efficient way to ameliorate excessive osteoclast mediated bone erosion has attracted more attention.

Osteoclasts were derived from haematopoietic stem cells (HSCs), which were differentiated by macrophage colony stimulating factor (M-CSF) and receptor activator of NF-κB ligand (RANKL) [5]. The binding of RANKL to RANK triggered the activation of nuclear factor-kappa B (NF-κB), mitogen-activated protein kinases (MAPKs), signal transducer and activator of transcription 3 (STAT3) and other classical signaling pathways to induce osteoclastogenesis [6]. c-Fos (a component of activator protein-1) acted as an essential transcription factor that initialed the expression of the nuclear factor of activated T-cells cytoplasmic1 (NFATc1) during osteoclast differentiation, proliferation and maturation [7]. Therefore, the osteoclastic specific marker genes such as cathepsin K (CTSK) and tartrate-resistant acid phosphatase (TRAP) were also up-regulated. In addition, monocyte/macrophage lineage cell-cell fusion was another unique process in osteoclastogenesis. During this process, CD9 played an essential role in regulating membrane fusion and the formation of multinucleated cells. In several inflammatory osteolysis diseases, excessive osteoclast fusion and bone resorption activity were also considered as significant elements to result in bone loss.

Lipopolysaccharide (LPS) was an important component of the outer membrane of gram-negative bacteria that contributed to the production and release of many pro-inflammatory cytokines to initiate inflammation [8]. During many studies on bone infection, LPS was reported to be the most vital regulator in inflammatory osteolysis [9]. Many experiments have also shown that LPS could enhance osteoclast differentiation mainly by increasing the expression of RANKL, which was highly dependent on the phosphorylation and nuclear translocation of NF-κB p65 [9]. Subsequently, the phosphorylation of STAT3 through direct (PI3K/Akt) or indirect (CD9/gp130/STAT3) pathways was also activated by stimulation with LPS alone [10]. The NF-κB and STAT3 signaling pathways have been shown to play essential roles in the osteoclast fusion, differentiation and bone resorption [11]. STAT3 signaling pathway was regulated by the activation of PI3K-Akt in cell proliferation and cell differentiation [12]. A recent study reported that LPS induced osteoclastogenesis via the activation of NF-κB-NFATc1 and STAT3 signaling pathways, which were significantly blocked by treatment with diallyl disulfide [13].

Epothilone B (Epo B) was an antineoplastic agent that was known to induce α-tubulin polymerization and enhance microtubule stability [14]. Administration of Epo B has been widely investigated for its neuroprotective effects via anti-inflammatory activity and inhibitory effect on fibrotic scarring after spinal cord injury by increasing fibroblasts apoptosis [15]. In particular, Epo B also showed protective effect in Parkinson’s disease through mediating microglial polarization via down-regulating the inflammatory stimulation [16]. However, the effects of Epo B on bone lytic diseases and osteoclast activity have not been reported.

Our study detected the inhibitory effects of Epo B on RANKL or LPS-induced osteoclastogenesis *in vitro* and the protective effects against LPS induced inflammatory femur osteolysis *in vivo*. Mechanistically, Epo B suppressed osteoclastogenesis by mediating direct or indirect STAT3 signaling to regulate the NF-κB signaling pathway. Additionally, the release of pro-inflammatory cytokines (IL-6, IL-1β and TNF-α) and the production of nitric oxide were significantly restrained. These suggested that Epo B administration acted as an effective strategy to treat inflammation-induced osteolysis due to its anti-inflammatory activity.

## RESULTS

### The cell viability, cell apoptosis rate and cell cycle were evaluated after treating with Epo B

**Figure 1:**
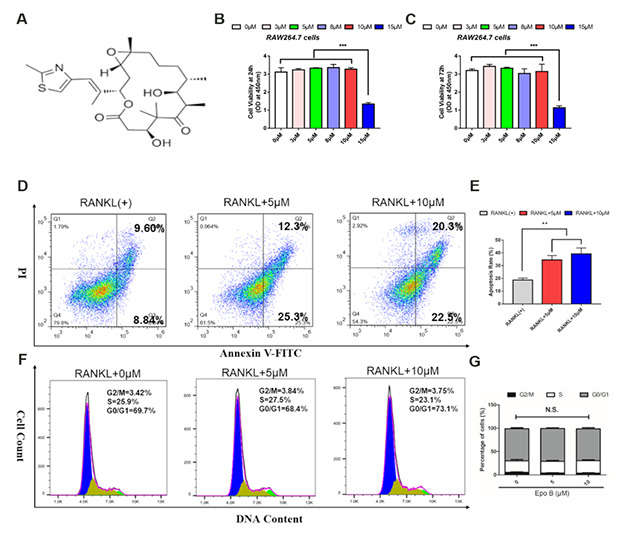
**Epothilone B dominantly induced osteoclast apoptosis without cytotoxicity.** (**A**) Chemical structure of Epothilone B. (**B–C**) CCK8 analysis of cell viability of RAW264.7 cells treated with different concentrations of Epothilone B (0μM to 15μM containing 0.1% DMSO) for 24 h or 72 h. (**D–E**) Flow cytometry analysis of osteoclast apoptosis during osteoclastogenesis in presence or absence of various dosage of Epothilone B (0μM, 5μM and 10μM). (**F–G**) Flow cytometry analysis of cell cycle during osteoclast differentiation by administration with or without Epothilone B (5μM and 10μM). Data in the figures represent mean ± SD. N.S. represented no significant difference. *p < 0.05, **p < 0.01, ***p < 0.001 based on one way ANOVA.

The chemical formula of Epo B was shown in Figure 1A. We first evaluated the cytotoxicity of Epo B on RAW264.7 cells for 24 h and 72 h, and found that Epo B at a low dose (< 10μM) had no cytotoxicity (Figure 1B, 1C). As many papers have noted that LPS at less than 100 ng/ml made no cytotoxicity [17]. Thus, 100 ng/ml LPS was chosen for further investigation. Additionally, although Epo B promoted α-tubulin polymerization and microtubule stability, low and high doses of Epo B did not make an apparent effect in regulating cell arrest at the G1 phase without cytotoxicity, which illustrated that the cell cycle of osteoclasts was not significantly influenced by Epo B (Figure 1F–1G). Since Epo B showed no significant difference in cytotoxicity analysis and cell cycle, we further detected the cell apoptosis at the same concentration of Epo B during osteoclastogenesis. Epo B at concentrations of 5 μM and 10 μM induced mature osteoclast apoptosis during osteoclastogenesis which was reflected by the increased apoptosis rate (Figure 1D, 1E).

### Epo B suppressed RANKL and LPS induced osteoclast differentiation

**Figure 2:**
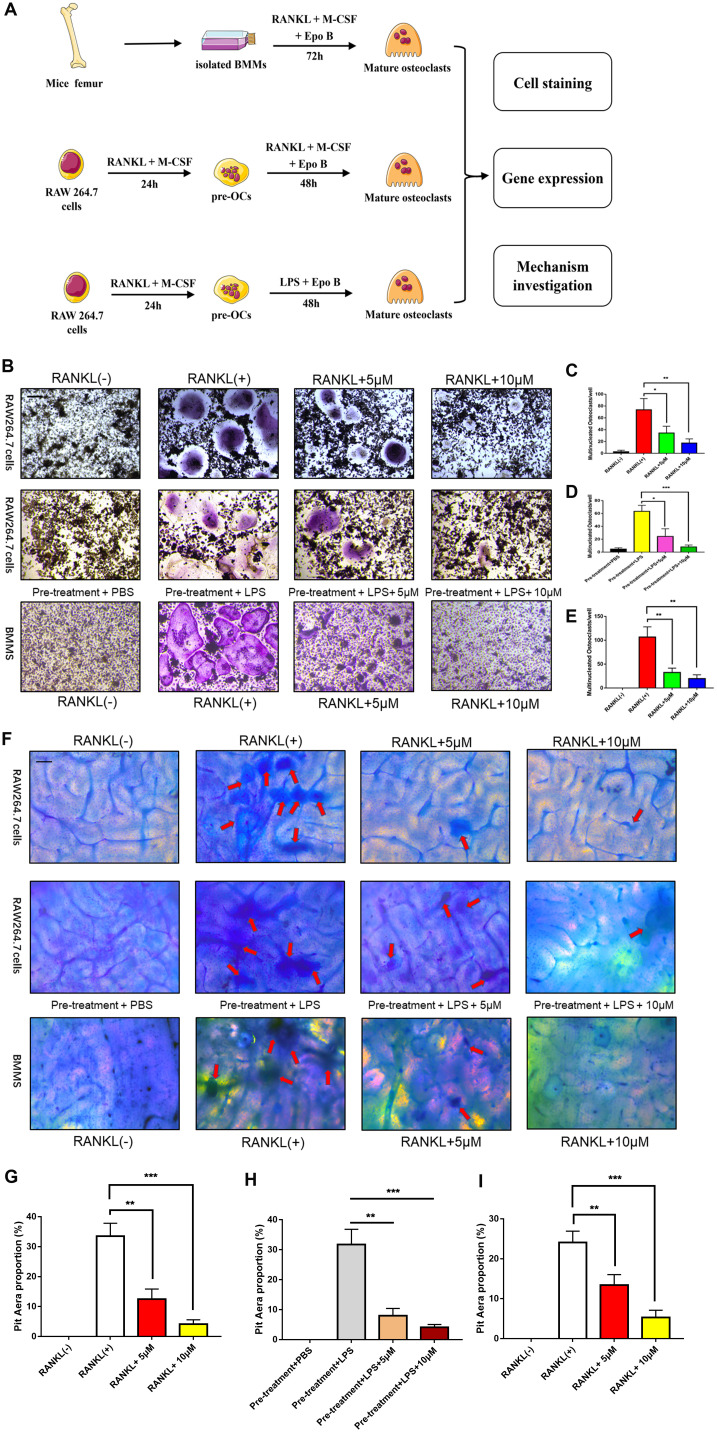
**Epothilone B inhibited RANKL or LPS induced osteoclast differentiation and bone resorption activity in a dose dependent manner.** (**A**) Schematic representation of the design of *in vitro* and *in vivo* experiments. (**B**) Representative images of RAW264.7 cells stained for TRAP treated with RANKL (100 ng/ml) and M-CSF (50 ng/ml) for 3 days or pretreatment with RANKL for 24h and LPS for another 48h. BMMs were incubated with RANKL (100 ng/ml) and M-CSF (50 ng/ml) for 5 days. All groups were incubated in the presence or absence Epothilone B. Scale bar = 200 μm. (**C**–**E**) Quantification of TRAP positive multinucleated osteoclasts (nuclei≥3) per well. (**F**) Representative images of RAW264.7 cells and BMMs were seeded onto bovine slices, which were also incubated with the same strategies. Scale bar = 200 μm. (**G**–**I**) Quantitative analysis of the osteoclastic bone resorption of bovine slices. Data in the figures represent mean ± SD. *p < 0.05, **p < 0.01, ***p < 0.001 based on one way ANOVA.

To detect the effect of Epo B on RANKL and LPS induced osteoclast differentiation, osteoclast specific TRAP staining was performed. Based on the cytotoxicity analysis of Epo B, 5 μM and 10 μM of Epo B were chosen to perform this staining. TRAP-positive (red) cells with more than three nuclei were counted as osteoclasts. Quantitative analysis of the results revealed that treatment with RANKL alone could dramatically promote osteoclastogenesis (Figure 2B–2E). Interestingly, RANKL-stimulated activation of osteoclastogenesis was dramatically reversed in the presence of 5 μM Epo B. After treatment with Epo B at 10 μM, mature osteoclasts with more than three nuclei were not found. Coincidently, LPS has been reported many times to play an essential role in inducing osteoclast differentiation. Treatment with LPS (100 ng/ml) alone for 72 h could not induce the mature osteoclasts. However, pre-treatment with RANKL (100 ng/ml) and M-CSF (50 ng/ml) for 24 h and then treatment with LPS for another 48h resulted in the activated multinucleated mature osteoclasts. When Epo B was added to the incubation system, osteoclast differentiation and TRAP activity were also dramatically abrogated (Figure 2B). In accordance, changes in mature osteoclast differentiation specific genes at the mRNA level were also detected. *c-Fos, NFATc1, TRAP, DC-STAMP, ATP6V0d2* and *OSCAR* were significantly suppressed by Epo B treatment during RANKL and LPS induced osteoclastogenesis, which were consistent with the TRAP staining results (Figure 4D). Moreover, Epo B also regulated the expression of genes in the early stage of osteoclast differentiation from monocytes to preosteoclasts such as CD9, mitf, c-Fos and NFATc1 (Figure 4E).

**Figure 3:**
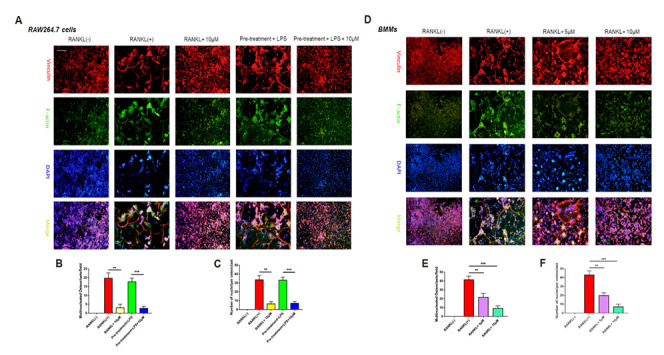
**Epothilone B made an inhibitory effect on osteoclast fusion significantly.** (**A**) Representative images of FAK staining of RAW264.7 cells induced by RANKL or LPS with or without Epothilone B treatment. Scale bar = 200 μm. (**B**–**C**) Quantitative analysis of multinucleated osteoclasts (nuclei≥3) and average the number of multinucleated osteoclasts. (**D**) Representative images of FAK staining of BMMs induced by RANKL in the presence or absence of various concentrations of Epothilone B. Scale bar = 200 μm. (**E**–**F**) Quantitative analysis of multinucleated osteoclasts (nuclei≥3) and average the number of multinucleated osteoclasts. Data in the figures represent mean ± SD. *p < 0.05, **p < 0.01, ***p < 0.001 based on one way ANOVA.

**Figure 4:**
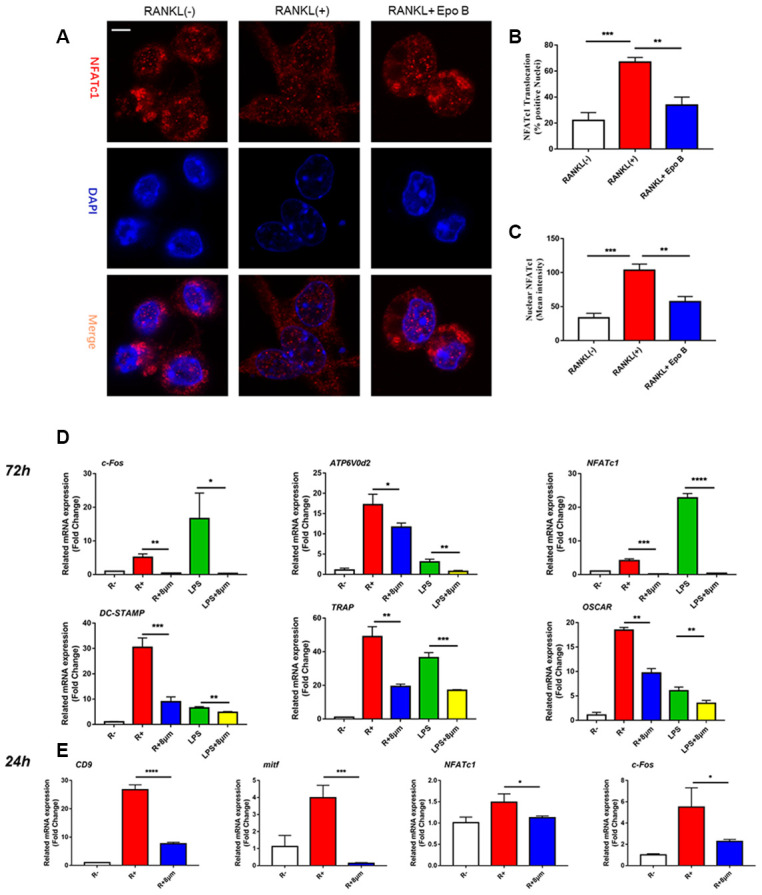
**Epothilone B suppressed NFATc1 nuclear translocation and the expression of marker genes during osteoclastogenesis.** (**A**) Representative images of immunofluorescence staining of the nuclear translocation of NFATc1 in the absence of presence of Epothilone B. Scale bar = 800 μm. (**B**) Quantitative analysis of the percentage of positive cells (NFATc1 translocation from cytosol to nuclear) in all cells. (**C**) Quantitative analysis of the mean intensity of NFATc1 in the cells nuclear. (**D**) Relative expression of marker genes in the procedure of osteoclastogenesis from monocytes to mature osteoclasts on mRNA level. (**E**) Relative expression of marker genes in the early stage of osteoclastogenesis on mRNA level.

**Figure 4:**
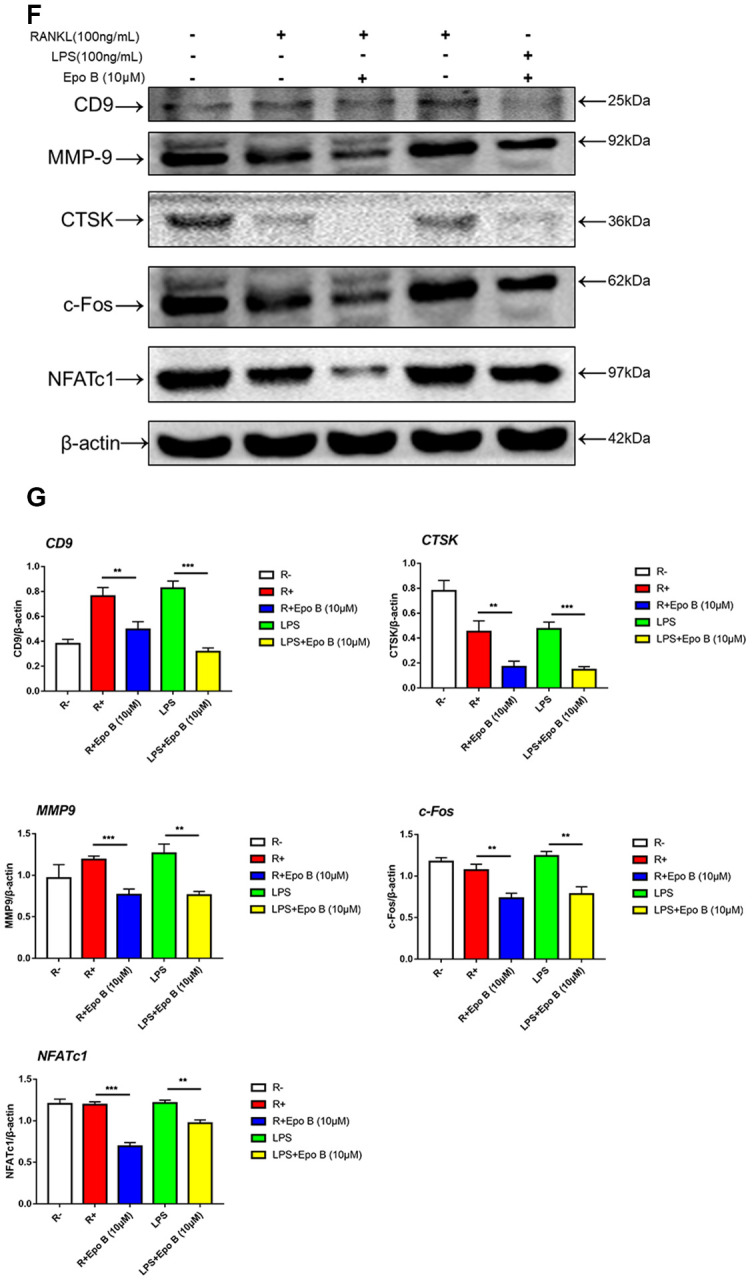
**Epothilone B suppressed NFATc1 nuclear translocation and the expression of marker genes during osteoclastogenesis.** (**F**) Relative expression of marker genes in the procedure of RANKL or LPS induced osteoclastogenesis on protein level. (**G**) Quantification of CTSK, MMP9, c-Fos, NFATc1 and CD9 relative to β-actin. Data in the figures represent mean ± SD. N.S. represented no significant difference. *p < 0.05, **p < 0.01, ***p < 0.001 based on one way ANOVA.

### Epo B attenuated F-actin ring formation and bone resorption activity of mature osteoclasts

To further detect the effect of Epo B on F-actin ring formation, we performed actin cytoskeleton and focal adhesion (FAK) staining to visualize the formation of the F-actin ring and cytoskeleton, respectively. The results showed that F-actin ring formation was dramatically inhibited in the presence of Epo B, which was consistent with TRAP staining results (Figure 3). Additionally, the average number of nuclei in mature osteoclasts was significantly decreased by Epo B treatment (Figure 3). These results indicated that osteoclast fusion was inhibited by Epo B administration.

Because the differentiation and fusion of osteoclasts were suppressed, bone resorption activity was also investigated to confirm whether the bone resorption activity of osteoclasts was influenced by Epo B. RAW264.7 cells and BMMs were seeded onto bovine bone slices according to the group settings. After incubation for 5 days, the cells were removed and the resorption area was quantified by using Image J software. Interestingly, the results showed that the erosion area was smaller in the Epo B-treated group than in the groups that were not treated with Epo B, which demonstrated that Epo B also strongly inhibited osteoclast bone resorption activity (Figure 2F–2I).

### Epo B restrained the release of pro-inflammatory cytokines and the production of nitric oxide

**Figure 5:**
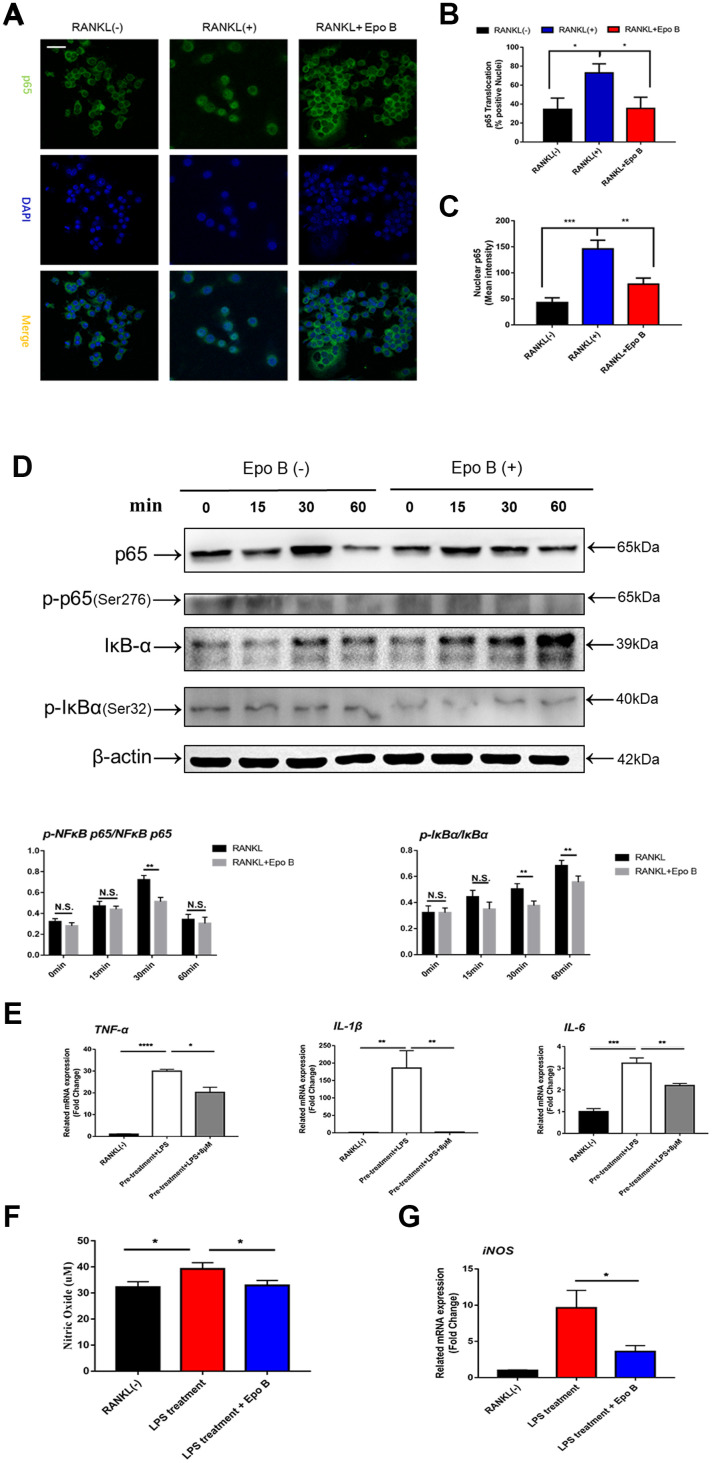
**Epothilone B restrained NF-ĸB signaling pathway and inhibited the release of pro-inflammatory cytokines and nitric oxide.** (**A**) Representative images of immunofluorescence staining of the nuclear translocation of NF-ĸB p65 in the absence of presence of Epothilone B. Scale bar = 400 μm. (**B**) Quantitative analysis of the percentage of positive cells (NF-ĸB p65 translocation from cytosol to nuclear) in all cells. (**C**) Quantitative analysis of the mean intensity of NF-ĸB p65 in the cells nuclear. (**D**) RAW264.7 cells were stimulated with RANKL with or without Epothilone B (8μM) for the 0-60 minutes. The cell lysates were analyzed using western blotting for p-NFκB p65, NFκB p65, p-IκBα and IκBα. Quantification protein expression of p-NFκB p65 relative to NFκB p65 and p-IκBα relative to IκBα. (**E**) Relative expression of pro-inflammatory cytokines (TNF-α, IL-1β, IL-6) during LPS induced osteoclast differentiation on mRNA level. (**F**) The concentration of NO was detected in the process of LPS-induced osteoclastogenesis. (**G**) Relative expression of iNOS (inducible nitric oxide synthetase) during LPS induced osteoclastogenesis on mRNA level. Data in the figures represent mean ± SD. N.S. represented no significant difference. *p < 0.05, **p < 0.01, ***p < 0.001 based on one way ANOVA.

Due to the anti-inflammatory effect of Epo B, the release of pro-inflammatory cytokines was also detected by using qRT-PCR. RAW264.7 cells were pre-treated with RANKL and M-CSF for 24 h and then stimulated with LPS. Treatment with LPS strongly promoted the release of pro-inflammatory cytokines and nitric oxide in the cytoplasm. Administration of Epo B dramatically reversed this promoted effect. Quantitative analysis showed that the mRNA levels of IL-6, IL-1β and TNF-α were also suppressed after treatment with Epo B compared to those of the positive control group (Figure 5E). We also tested the concentration of NO in the cell culture supernatant during LPS-induced osteoclast differentiation. Epo B treatment evidently decreased NO production compared with that of the control group (Figure 5F). Interestingly, Epo B inhibited the expression of inducible nitric oxide synthase (iNOS) at the mRNA level, which was consistent with suppressive effect of NO production after administration of Epo B (Figure 5G). Overall, Epo B played an inhibitory role in the release of pro-inflammatory cytokines and the production of NO.

### Suppression of NF-κB signaling pathway attenuated the activated expression of NFATc1

Due to the excellent anti-osteoclastic effect of Epo B during osteoclastogenesis, the potential mechanism was detected. RANKL stimulated NF-κB signaling pathway was an essential step in osteoclast differentiation. After stimulating RAW264.7 cells with RANKL could significantly enhance NF-κB p65 activity, the phosphorylation of NF-κB p65, the phosphorylation and degradation of IκB when treated for 60 min (Figure 5D). However, these effects were significantly reversed after co-culturing with Epo B. To determine whether the nuclear translocation of NF-κB p65 was influenced by Epo B, immunofluorescence analysis was performed. Treatment with only RANKL dramatically enhanced the nuclear translocation of NF-κB p65. In addition, the mean intensity of NF-κB p65 in the nuclei and the number of cells with positive nuclear translocation were robustly suppressed by treatment with Epo B (Figure 5A–5C). Together, these results indicated that Epo B attenuated RANKL induced osteoclastogenesis through regulating NF-κB signaling pathway.

The inhibitory effects of Epo B on osteoclast related signaling pathways contributed to the regulation of master factors such as NFATc1 and c-Fos, which were evaluated by western blotting. Epo B significantly downregulated the protein expression of NFATc1, c-Fos, CTSK and TRAP during the procedure of RANKL and LPS induced monocytes to mature osteoclasts (Figure 4F). Because of the dramatic suppression of NFATc1, whether Epo B inhibited the nuclear translocation of NFATc1 was also investigated. Coincidently, as the data (Figure 5A–5C) showed that treatment with Epo B repressed the mean intensity of NFATc1 in the nuclei of osteoclasts and the number of nuclei translocation positive cells. Taken together, these data confirmed that Epo B attenuated osteoclast fusion and differentiation by regulating NF-κB signaling pathway to influence the expression of osteoclastic marker genes.

### Epo B abrogated the activation of direct or indirect STAT3 signaling pathway to negatively regulate the activation of NF-κB

**Figure 6:**
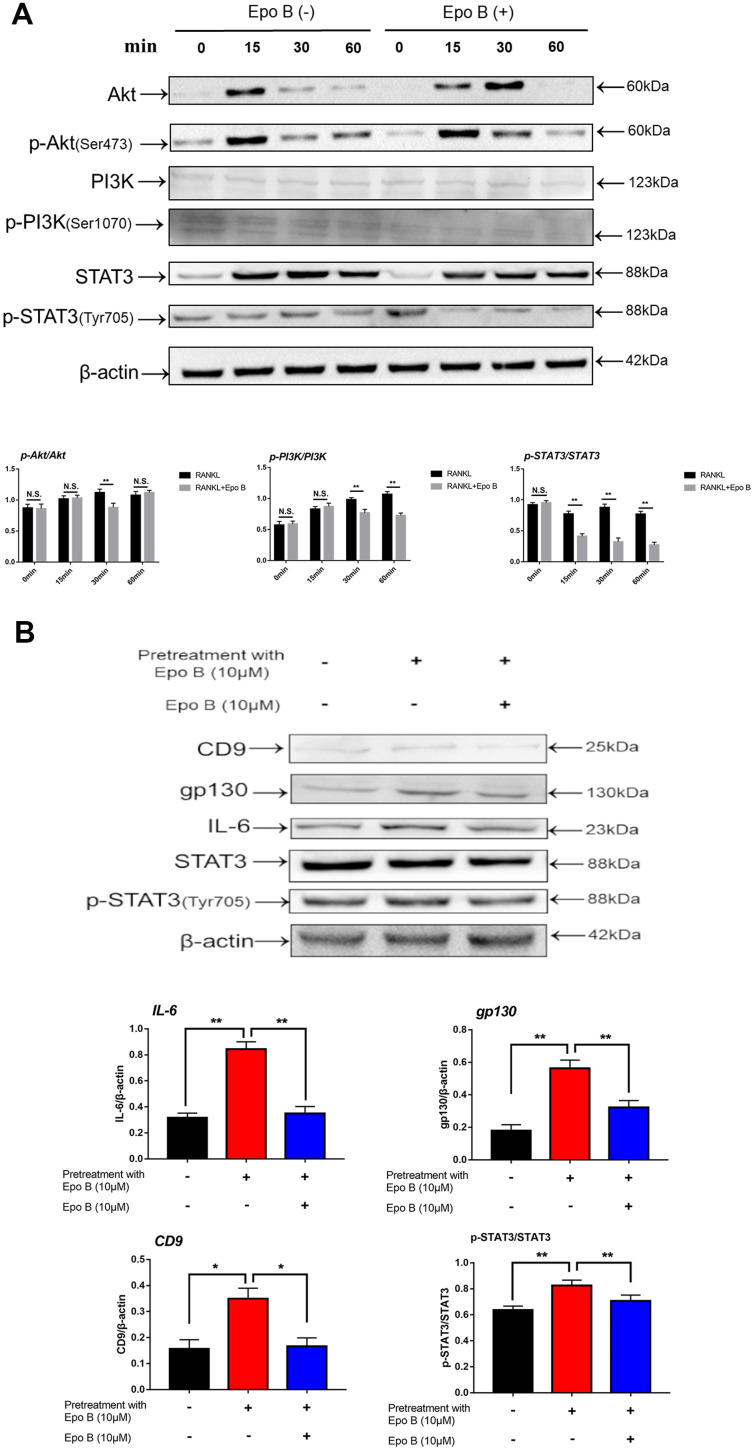
**Epothilone B significantly restrained STAT3 signal pathway both in direct and indirect manner.** (**A**) RAW264.7 cells were stimulated with RANKL (100ng/mL) with or without Epothilone B (10μM) for the 0-60 minutes. The expression of p-Akt, Akt, p-PI3K, PI3K, p-STAT3 and STAT3. (**B**) RAW264.7 cells were divided into 3 groups. One group was pretreated with RANKL (100ng/mL), M-CSF (100ng/mL) and LPS (100ng/mL) in presence of Epothilone B (10μM) for 24 h. The other two groups were pretreated with RANKL (100ng/mL), M-CSF (100ng/mL) and LPS (100ng/mL) but in absence of Epothilone B (10μM) for 24 h. After that, one group was treated with vehicle, and the others were incubated with Epothilone B (10μM) for 2 h. Next, all groups were treated with RANKL (100ng/mL) for 30 min. The cell lysates were analyzed by Western blot for CD9, IL-6, gp130, STAT3, p-STAT3. β-actin was used as an internal control. Quantification of IL-6, gp130 and CD9 relative to β-actin, and p-STAT3 relative to STAT3. Data in the figures represent mean ± SD. N.S. represented no significant difference. *p < 0.05, **p < 0.01, ***p < 0.001 based on one way ANOVA.

In addition to activation of the NF-κB signaling pathway played an important role in osteoclastogenesis during inflammation, STAT3 signaling also participated in this process. STAT3 could induce NF-κB p65 activity, whose activation contributed to the transcriptional regulation of inflammatory cytokines, such as IL-1β, IL-6 and TNF-α [18]. The direct and indirect STAT3 signaling pathways were also detected by using western blotting and qPCR. Western blot analysis was used to detect changes in the expression of p-STAT3 directly and indirectly after treatment with Epo B. RAW264.7 cells were pre-treated with vehicle or Epo B (8 μM) for 2 h followed by stimulation with RANKL (100 mg/ml) for 0, 15, 30, or 60 min. The results revealed that RANKL stimulated the Tyr705 phosphorylation of STAT3 was markedly attenuated by treatment with Epo B directly when the incubation lasted for 30 min (Figure 6A).

PI3K/Akt signaling also regulated osteoclast fusion and differentiation [19]. Upon RANKL stimulation, activated PI3K promoted Akt phosphorylation at Ser473 and subsequently activated the downstream Akt pathway. Moreover, many previous studies have revealed that the PI3K/Akt axis played an important role in the activation of STAT3 [20]. Since the STAT3 signaling pathway was significantly inhibited, we investigated whether Epo B could mediate the suppression of osteoclastogenesis by phosphorylation of STAT3 via the PI3K/Akt axis. Our results showed that Epo B inhibited the phosphorylation of PI3K at Ser1070 and Akt at Ser473 in a time-dependent manner, which was similar to reports that blocking PI3K/Akt signaling pathway dramatically inhibited p-STAT3 (Figure 6A). These results suggested that inhibition of the PI3K/Akt pathway restrained the phosphorylation of STAT3 as well as regulated the osteoclastogenesis.

Moreover, CD9 played a critical role in regulating osteoclast fusion during the early stage. Interleukin-6 (IL-6) receptor subunit gp130 (glycoprotein 130) was a potential CD9-interacting protein. Once IL-6 combined with the IL-6 receptor, gp130 dimerized and triggered signal transduction to induce phosphorylation (p-Tyr705) of STAT3, resulting in the nuclear translocation of STAT3 and promoting the transcription of STAT3 downstream genes. Epo B treatment significantly reduced the expression of CD9 during early osteoclastogenesis compared with that of treatment with RANKL alone, which indicated that the early fusion of OCs was inhibited by treatment with Epo B. Due to the potential mechanism of this inhibitory effect of CD9, we hypothesized that Epo B indirectly regulated the CD9/gp130/STAT3 signaling pathway indirectly. We pretreated RANKL and M-CSF with or without Epo B for 24 h, and the culture medium was replaced with DMEM without FBS for 12 h to maintain an equal level of phosphorylation among the different groups. In addition, these groups were also incubated with vehicle or Epo B followed by RANKL and M-CSF for another 60 min. Then the expression of IL-6, gp130, total STAT3 and phosphorylated (Tyr705) STAT3 were detected by using western blotting. Epo B dramatically inhibited the expression of IL-6 and gp130, which contributed to suppress the phosphorylation (Tyr705) of STAT3. These results suggested that Epo B reduced the phosphorylation (Tyr705) of STAT3 directly and inhibited the binding of CD9 and gp130 to make a suppressive effect on the phosphorylation (Tyr705) of STAT3 indirectly (Figure 6B). Collectively, Epo B dramatically inhibited the activation of STAT3 through direct or indirect mechanisms during RANKL stimulation.

### Epo B protected LPS-induced inflammatory bone destruction *in vivo*

**Figure 7:**
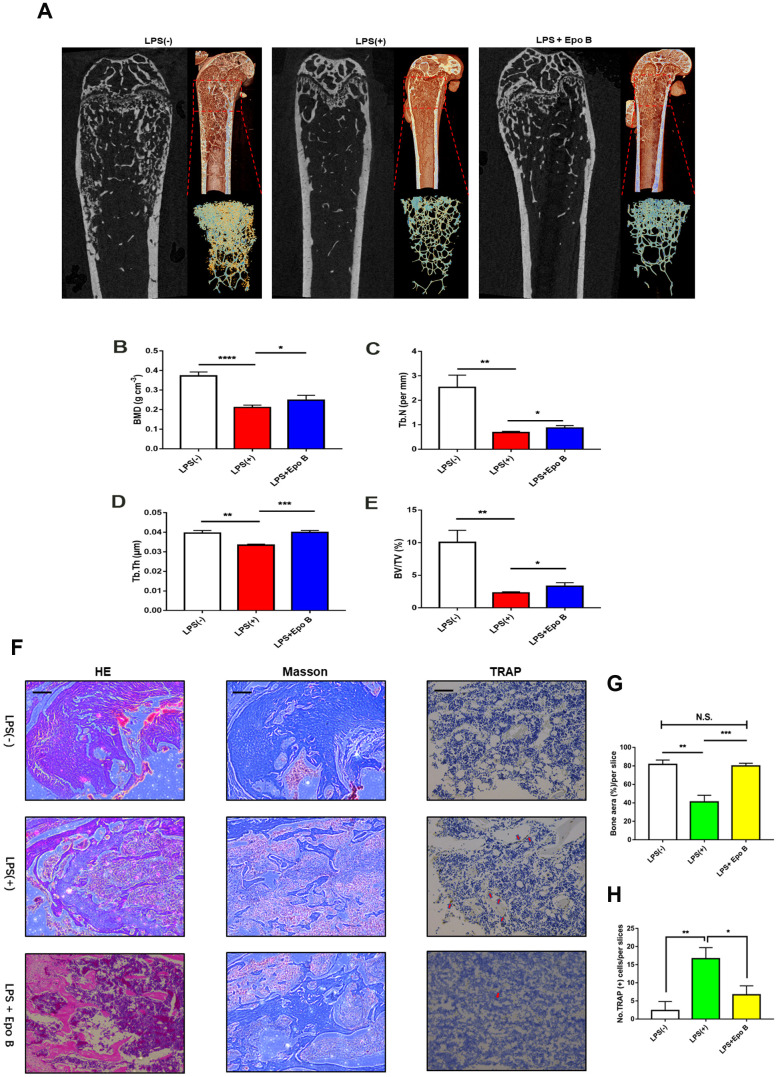
**Epothilone B prevented LPS-induced inflammatory osteolysis *in vivo*.** (**A**) Representative μCT longitudinal section images of the femurs, cross-sectional view of the distal femurs and reconstructed trabecular structure of the ROI (red dashed box). (**B**–**E**) Quantitative μCT analysis of distal femoral volumetric bone mineral density (BMD), BV/TV, trabecular thickness (Tb.Th) and trabecular number (Tb.N) in each group. (**F**) Representative images of histological slides of HE stain, Masson stain and immunohistological stain for TRAP focusing on the metaphyseal region of the distal femur from mice of different groups. Scale bar = 800 μm (**G**) Quantitative analysis of the percentage of bone area in each slice which was reflected by HE staining. (**H**) Quantitative analysis of the number of TRAP positive cells in each slice which was reflected by immunohistological stain for TRAP. Data in the figures represent mean ± SD. *p < 0.05, **p < 0.01, ***p < 0.001 based on one way ANOVA.

**Figure 8:**
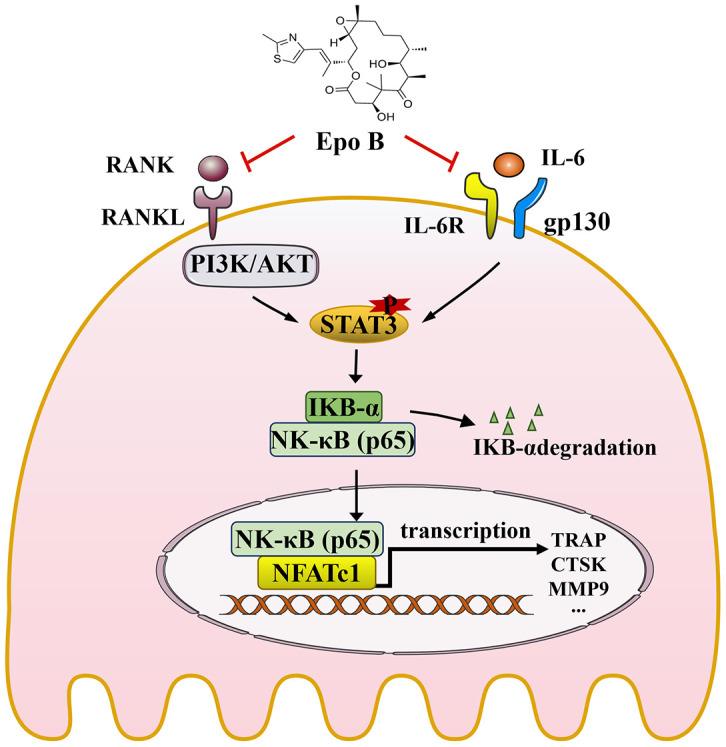
**Schematic diagrams showing the potential mechanism in the protective effects of Epothilone B on LPS induced inflammatory bone destruction.**

To evaluate the effect of Epo B on LPS induced inflammatory osteolysis *in vivo*, we have established LPS-induced inflammatory bone destruction mouse model. LPS was injected intraperitoneally alone or in combination with Epo B for 6 weeks. μCT analysis was performed to detect the bone mineral density (BMD), trabecular bone volume fraction (BV/TV), trabecular thickness (Tb.Th) and trabecular number (Tb.N) of the distal femur. The results showed that the BMD, BV/TV, Tb.Th and Tb.N were significantly increased after administration with Epo B in the LPS induced bone-loss mouse model compared with those of treatment with LPS alone (Figure 7A–7E). Histological analysis showed that IHC staining for TRAP was dramatically inhibited by treatment with Epo B compared with that of LPS treatment. The TRAP positive cells were observed in the LPS-induced group relative to those of the Epo B groups and were markedly reduced (Figure 7H). Moreover, HE staining results showed that administration with Epo B inhibited the loss of bone area, which was consistent with the micro-CT imaging of bone parameters (Figure 7G). Additionally, Masson staining results revealed that injection of Epo B significantly improved the destruction of collagen during inflammatory osteolysis. Taken together, these animal experiments results confirmed that Epo B protected inflammatory bone loss *in vivo* and might serve as an important anti-resorptive agent to prevent inflammatory osteolysis diseases (Figure 8).

## DISCUSSION

Osteomyelitis was an essential part of bone infection disease and characterized by a severe inflammatory reaction that contributed to bone destruction due to the release of pro-inflammatory cytokines (TNF-α, IL-1β and IL-6) from many bacterial [21]. The most highly virulent bacteria in the early and acute states of osteomyelitis was *S. aureus*, which entered the bone via the bloodstream [2]. LPS, a bacterial endotoxin that was a component of *S. aureus* initiated the recruitment of immune cells during inflammatory osteolysis and further induced the secretion of pro-osteoclastogenic cytokines [22]. Therefore, many studies have shown that LPS was the most widely known microbial-associated molecular pattern that was used to establish inflammatory bone destruction murine model [23]. Osteoclasts were considered as essential type of cells in the skeletal system and were derived from monocyte/macrophage progenitor cells or haematopoietic stem cells. RANKL and M-CSF were indispensable and exclusive cytokines for osteoclast fusion and differentiation. In this study, we investigated whether Epo B prevented LPS-induced osteolysis *in vivo* by regulating osteoclastogenesis, which was mediated by suppression of the STAT3-dependent signaling pathway.

Epo B was an FDA-approved, anti-neoplastic drug that has been used clinically in the treatment of ovarian cancer [24]. Several reports have demonstrated that anti-inflammatory effects in the brains of Parkinson’s disease patients may further exacerbate neurodegeneration [25]. Moreover, Epo B treatment inhibited the increased levels of TNF-α, IL-1β and IL-6 in 6-OHDA-stimulated BV2 cells that were cocultured with MN9D neurons [26]. The present study was designed to determine whether Epo B exerted inhibitory effects on osteoclastogenesis and prevented the LPS induced inflammatory bone loss.

Excessive osteoclasts contributed to the imbalance in bone homeostasis in inflammatory osteolysis and the development of potential therapies were needed. In the present study, Epo B inhibited osteoclast fusion and differentiation which was reflected by the reduction of the number of mature multinucleated osteoclasts. Additionally, the results also showed the suppressive effect of Epo B on the bone resorption of mature osteoclasts, which indicated that the function of mature osteoclasts was also inhibited by Epo B administration during RANKL and LPS induced osteoclastogenesis. We also detected that treatment with Epo B suppressed the mRNA expression of NFATc1, c-Fos, TRAP, ATP6V0d2, DC-STAMP, and OSCAR during osteoclast differentiation in the early and late stages. IL-1β, IL-6 and TNF-α acted as powerful inducers of osteoclastogenesis and promoted the bone resorption after stimulation with RANKL through MAPKs and NF-κB signaling. Moreover, iNOS played an essential role in the generation of NO, which also promoted the formation of multinucleated osteoclasts through many processes such as cell fusion of osteoclast precursors and increased actin remodeling in pre-OCs. In detail, LPS could induce the increased levels in IL-1β, IL-6, TNF-α, NO and iNOS, which were reversed by Epo B treatment. Furthermore, Epo B administration significantly reduced the number of TRAP-positive cells *in vivo* when compared with that of the LPS group. HE and Masson staining further confirmed that the protective effect of Epo B on LPS stimulated bone erosion was accompanied by the inhibitory effect of osteoclast fusion and differentiation. Additionally, the micro-CT analysis showed that Epo B treatment significantly mitigated the severity in bone erosion of the LPS-induced osteolysis model, as illustrated by an increased level in BMD, BV/TV, Tb.N, and Tb.Th. Taken together, the current study was the first time to show that the protective effect of Epo B on inflammatory bone loss was based on inhibition of RANKL or LPS induced osteoclastogenesis *in vitro* and its protective effect on inflammatory osteolysis *in vivo.*

RANKL-induced NF-κB and STAT3 signal pathway activation were essential for osteoclast fusion, differentiation and bone resorption activity. However, no research has demonstrated that these two pathways participated in the Epo B stimulation. In addition, STAT3-related signaling pathways also played pivotal roles in osteoclastogenesis by acting as essential regulators of the NF-κB activity. In the present study, we demonstrated that Epo B made an inhibitory effect on RANKL or LPS induced osteoclast fusion and differentiation via direct or indirect suppression of the PI3K/Akt/STAT3 signaling pathways due to its anti-inflammatory effect, which was consistent with the protective effect of Epo B on inflammatory osteolysis *in vivo*. Accumulating evidence has confirmed that the binding of RANKL to its receptor RANK on osteoclast precursor cells activates downstream signaling pathways, including the NF-κB pathway during osteoclastogenesis [27]. Studies of the underlying mechanism have revealed that inactive NF-κB subunits are retained in the cytoplasm by inhibitory IκB. The activated IKK complex catalyses the phosphorylation and subsequent degradation of IκB, which releases NF-κB p65/Rel A to translocate to the nucleus and initiate target gene transcription [28, 29]. Moreover, previous studies have revealed that activation of STAT3 pathway was a crucial promoter in osteoclast formation and inflammatory bone loss [30]. STAT3 played a predominant role in several cellular events in osteoclastogenesis, acting as a crucial transcription factor to regulate the expression of CTSK, CTR, TRAP, MMP9 and the release of many pro-inflammatory cytokines in an NF-κB dependent manner [31, 32]. STAT3 phosphorylation was directly suppressed by dramatically repressing PI3K/Akt after Epo B treatment. In addition, CD9 made functions by regulating gp130 protein stability to maintain the self-renewal of cell differentiation [33]. CD9 acted as a cell surface protein and had been reported to be a significant fusogenic gene in maintaining the fusion of osteoclasts [34]. CD9 stabilized the IL-6 receptor subunit gp130, which in turn promoted the activation of STAT3 phosphorylation [35]. Because CD9 was preferentially expressed in mature osteoclasts, dramatically inhibited CD9 expression may specifically disrupt the formation of osteoclasts, driving excessive bone resorption associated inflammatory osteolysis. Generally, pretreatment with Epo B revealed that targeting CD9 to interrupt the gp130-STAT3 signaling axis may also inhibit the expression of osteoclastic genes, which may help us to develop potential therapeutics to improve inflammatory osteolysis treatment.

Although the inhibitory effect of Epo B on excessive bone resorption activity was detected, there were also several limitations worthy of further investigation. First, the interaction between osteoclast-mediated bone resorption and osteoblast-mediated bone formation also played vital roles in the treatment of inflammatory bone destruction. Therefore, osteoblastic bone formation and the possible mechanism also need to be evaluated after treatment with Epo B. As the undesirable status was completely reversed, the inhibition of bone destruction by Epo B was thought to be mainly due to its suppression of osteoclastogenesis. Second, an initiating agent of osteolysis other than LPS should also be administered to further confirm the beneficial effect of Epo B. Third, the regulation of many immunocytes during the treatment of inflammatory osteolysis with Epo B was also needed to be confirmed.

In summary, our study demonstrated that Epo B suppressed osteoclastogenesis and osteoclast bone resorption activity *in vitro* and LPS-induced inflammatory bone destruction *in vivo*. We also confirmed that the underlying mechanism of this inhibitory effect was direct suppression of the PI3K/Akt/STAT3 pathway or indirect suppression of the CD9/gp130/STAT3 pathway, thereby mediating nuclear translocation of NF-κB p65 to downregulate the master genes such as c-Fos and NFATc1 so as to regulate osteoclast differentiation and fusion. These results indicated that Epo B was a potential therapeutic agent that protected against inflammatory excessive osteoclast-related diseases such as chronic implant-related bone infections, osteomyelitis, periprosthetic infection and other inflammatory osteolysis.

## MATERIALS AND METHODS

### Cells, reagents and antibodies

Epothilone B (Epo B) was purchased from MedChemExpress (Princeton, NJ). Recombinant Mouse M-CSF and Recombinant Mouse RANKL were obtained from R&D Systems (Minneapolis, MN). TRAP stain kit and Lipopolysaccharide from Escherichia coli O55:B5 were purchased from Sigma-Aldrich (St. Louis, USA). Antibodies against c-FOS, NFATc1, IL-6 and gp130 were from Cell Signaling Technology Inc (Danvers, USA). Antibodies against phospho-NFκB p65, NF-κB p65, phospho-IκBα, IκBα, STAT3, phospho-STAT3 (Tyr705) and β-actin were from Bioworld Technology (St. Louis Park, MN, USA). Antibody against Akt, phospho-Akt (Ser473), PI3K, phospho-PI3K (Ser1070), MMP9, CD9 and CTSK were purchased from bioss (Beijing, China). Nitric oxide detection kit (Beyotime Biotechnology, Shanghai, China). CCK-8 kit was purchased from Dojindo Molecular Technologies (Tokyo, Japan). The Focal Adhesion and Actin Cytoskeleton Staining Kit was obtained from Millipore (Darmstadt, Germany). Bovine cortical bone slices for pit formation assay were obtained from Boineslices.com (Jelling, Denmark). Dulbecco’s modified eagle medium (DMEM), alpha minimal essential medium (α-MEM), and fetal bovine serum (FBS) were purchased from Gibco (Life Technologies, USA). RAW264.7 cells (mouse macrophage cells) were obtained from the American Type Culture Collection (Rockville, MD, USA).

### Cytotoxicity and flow cytometry assays

For selecting a range of suitable concentration of Epo B, cell counting kit-8 (CCK8) assay was used to assess cytotoxicity. RAW264.7 Cells were seeded onto 96 wells plate, the cytotoxicity of Epo B tested using concentration of 0μM to 15μM for 24h and 72h. The cells then were washed with PBS and incubated with serum free medium containing 10% CCK-8 reagent for 2 hours at 37°C in an atmosphere containing 5% CO2. The absorbance at 450nm was measured by using Bio-TEK, Synergy H4 Microplate reader.

Flow cytometry was used for analysis of cell cycle change and cell apoptosis. For detecting different stages distribution of cell division analysis, 1×10^6^ RAW264.7 cells were induced with RANKL (100 ng/ml) and M-CSF (50 ng/ml) for 72h with or without treatments of Epo B (5μM, 10μM). Cells were then washed in PBS for 2 times and fixed by 70% ethanol. After fixation, cells were washed with PBS for another time and stained with 10μg propidium iodide (PI, Sigma–Aldrich) together with RNase treatment for 30 min (37°C). A FACStar flow cytometer (BD, CA, USA) was adopted to measure the percentages of cells in the different cell cycle phases. The results were analyzed by Lysis II and Cellfit software (BD). Cell apoptosis was determined by Annexin V/PI staining as previously described. In brief, RAW264.7 cells were induced with RANKL (100 ng/ml) and M-CSF (50 ng/ml) for 72h with treatments of Epo B at different dosages (0, 5μM, 10μM). Cells were washed twice with cold PBS and then resuspended in 100 μl of binding buffer [10 mM HEPES/NaOH (pH 7.4), 140 mM NaCl, 2.5 mM CaCl2] at a concentration of 1×10^6^ cells/ml. Cells were then stained with 5 μl of annexin V-FITC (Life Technologies) and 10 μl of 20μg/ml PI. Apoptosis was analyzed using a FACStar flow cytometer (BD).

### *In vitro* osteoclastogenesis assays

Bone marrow monocytes/macrophages (BMMs) were separated from mouse femur and cultured with M-CSF (50ng/ml) for 24h to obtain BMMs. BMMs were cultured in α-MEM containing 10% FBS and 1% Penicillin streptomycin solution. For tartrate resistant acid phosphatase (TRAP) stain, RAW264.7 cells and BMMs were seeded onto 96-wells plate and incubated with RANKL (100ng/ml) and M-CSF (50ng/ml) for 3 or 5 days. What’s more, RAW264.7 cells were also seeded on 96-wells plate and incubated RANKL (100ng/ml) and M-CSF (50ng/ml) for 24h. Then the medium was replaced with PBS or LPS (100ng/ml), incubated for another 48h so as to complete the induction of osteoclast differentiation during inflammatory situation. All cells were fixed with 4% paraformaldehyde for 15 min after being washed twice with PBS and then stained with TRAP staining solution (0.1mg/ml of naphthol AS-MX phosphate, 0.3mg/ml of Fast Red Violet LB stain) on the basis of the manufacturer’s directions. Three or more nuclei multinucleated cells were defined mature osteoclasts and counted by using optical microscope (DMI 6000B; Leica Microsystems, Wetzlar, Germany).

For Actin Cytoskeleton and Focal Adhesion (FAK) stain, cells were fixed with 4% paraformaldehyde for 20 min and permeabilized with 0.1% Triton X-100 for 5 min at room temperature. After blocking, primary antibody (Anti-Vinculin) was then diluted to a working concentration (1:300) in blocking solution, and cells were incubated for 1 hour at room temperature. Secondary antibody (Alexa Fluor 488 Goat Anti-Mouse IgG (H+L) Antibody, Invitrogen) (1:500) and TRITC conjugated Phalloidin (1:500) was diluted in 1× PBS and cells were incubated for 1h at room temperature. Nuclei counterstaining was performed by DAPI (1:1000) for 5 minutes followed by fluorescence microscopy and confocal microscopy observation.

For resorption pit assay, BMMs and RAW264.7 cells were seeded onto bovine cortical bone slices and incubated with the same strategy. Cells were eliminated with sodium hypochlorite for 5 min at room temperature and then washed twice with distilled water. Many details were described in a previous report [36], and the absorption area was analyzed using the Image J software (NIH, Bethesda, MD).

### Confocal observation of p65 and NFATc1 nuclear translocation

For observing the effect of Epo B on the nuclear translocation of p65 and NFATc1 following RANKL stimulation, immunofluorescence staining was performed respectively. RAW264.7 cells were pretreated with 8μM Epo B for one hour followed by stimulation with 100 ng/ml RANKL for 30 min. Cells were fixed with 4% PFA, permeabilization and blocking with blocking buffer at room temperature for one hour at room temperature. Then cells were washed with PBS for three times and incubated with primary antibodies at 4°C overnight. The colocalization of p65 and NFATc1 antibodies were incubated with Alexa Fluor 647 or 488 secondary antibody (Abcam, Cambridge, United Kingdom) for one hour at room temperature in the dark. Nuclei were colored with DAPI for 5 min in the dark. Images were observed by using a confocal fluorescence microscopy (Leica, TCS-SP5, DM6000-CFS). The gray value was analyzed using the Image J software (NIH, Bethesda, MD).

### Nitric oxide detection

For detection the concentration of nitric oxide detection after treating with Epo B, many details were performed as previously described by previous study. RAW264.7 cells were seeded into 6-wells plate and pre-incubated with RANKL (100ng/ml) and M-CSF (50ng/ml) for 24h. Respectively, the medium was replaced with DMEM and LPS (100 ng/ml) containing or not containing Epo B for an another 48 h. The supernatant was centrifuged at 1000 rpm for 5 min to remove purify and added into 96-well plates. The reaction between the supernatant and nitric oxide (NO) Detection Kit (Beyotime Biotechnology, Shanghai, China) was performed according to the manufacturer’s instructions. The absorbency at 450nm was measured using Bio-TEK, Synergy H4 Microplate reader. Standard concentration gradient was used as a standard curve.

### Real-time reverse transcription polymerase chain reaction analysis

Total RNA was isolated from cultured cells using Trizol reagent (Thermo Fisher Scientific). cDNA was reversed from total RNA by using a Revert Aid First Strand cDNA Synthesis Kit (Thermo Fisher Scientific). The reverse transcription product was then used as a template to perform real-time PCR on a Step One Plus thermal cycler (Applied Biosystems) in SYBR Green Master Mix (Takara), and amplification was performed using an ABI Prism 7500 System (Thermo Fisher Scientific) following the manufacturer's guidelines. The primers were performed as Table 1.

**Table 1:** Primer sequences for qPCR.

**Genes**	**Forward**	**Reverse**
c-Fos	5′-CGGGTTTCAACGCCGACTA-3′	5′-TTGGCACTAGAGACGGACAGA-3′
mitf	5′-AACTCCTGTCCAGCCAACCTTC -3′	5′-TCTGCCTCTCTTTAGCCAATGC -3′
CD9	5'-CGGTCAAAGGAGGTAG-3'	5'-GGAGCCATAGTCCAATA-3'
TRAP	5'-CACTCCCACCCTGAGATTTGT-3'	5'-CATCGTCTGCACGGTTCTG-3'
OSCAR	5′-GGTCCTCATCTGCTTG-3′	5′-TATCTGGTGGAGTCTGG-3′
DC-STAMP	5′-CTAGCTGGCTGGACTTCATCC-3′	5′-TCATGCTGTCTAGGAGACCTC-3′
ATP6V0d2	5′-AGCAAAGAAGACAGGGAG-3′	5′-CAGCGTCAAACAAAGG-3′
NFATc1	5′-CCCGTCACATTCTGGTCCAT-3′	5′-CAAGTAACCGTGTAGCTGCACAA-3′
TNF-α	5′-AGGCGGTGCTTGTTCCTCA-3′	5′-AGGCGAGAAGATGATCTGACTGCC-3′
IL-1β	5′-CTCAACTGTGAAATGCCACC-3′	5′-TGTCCTCATCCTGGAAGGT-3′
IL-6	5′-TGGGAAATCGTGGAAATGAGA-3′	5′-ACTCTGGCTTTGTCTTTCTTGT-3′
iNOS	5'-GCAGAATGTGACCATCATGG-3'	5'-ACAACCTTGGTGTTGAAGGC-3'
β-actin	5′-TCCCTGTATGCCTCTG-3′	5′- ATGTCACGCACGATTT-3′

### Western blot analysis

Total protein was extracted from cultured cells by using radioimmunoprecipitation assay (RIPA) buffer containing phosphatase inhibitors, protease inhibitors, 50mmol/L Tris [Ph 8], 250 mmol/L NaCl, 0.05% sodium dodecyl sulphate, 0.5% deoxycholate and 1% NP-40. Proteins were separated using SDS-PAGE then transferred to PVDF membrane. Primary antibodies (phospho-NFκB p65, NF-κB p65, phospho-IκBα, IκBα, STAT3, phospho-STAT3 (Ser727), Akt, phospho-Akt (Ser473), PI3K, phospho-PI3K (Ser1070), CD9, CTSK, c-FOS, NFATc1, IL-6 and gp130) were incubated at 4°C overnight. Then the membranes were washed with TBST for three times and immersed in secondary antibody at room temperature for 2 hours. β-actin acted as loading control. Many details have been reported in our previous study [37].

### Inflammatory bone destruction model, micro-CT analysis and histological analysis

Mice for establishing inflammatory bone destruction model, 8 weeks female C57BL/6 mice were provided by the animal center of Army Medical University. All procedures involving mice and experimental protocols were performed according to the guidelines of laboratory animal care and approved by Army Medical University. Mice were divided into three groups: PBS treated group (n=5), LPS (8mg/kg) treated group (n=5), LPS (8mg/kg dissolved in saline) + Epo B (1mg/Kg dissolved in DMSO and saline at a ratio of 1:3) treated group (n=5). They were injected intraperitoneally second times a week for 6 weeks. Mice were weighed daily, and the required concentration of Epo B and LPS were calculated. All treated mice were killed by cervical dislocation one day after last administration.

For the micro-computed tomography (micro-CT) analysis, the femur tissue was separated from experimental mice. Bruker Micro-CT Skyscan 1272 system (Kontich, Belgium) with an isotropic voxel size of 10.0 μm was used to image the whole femur. Scans were conducted in 4% PFA and used an X-ray tube potential of 60kV, an X-ray intensity of 166μA, and an exposure time of 1700ms. The threshold for the trabecular and cortical bones was set at 86–255 (8-bit gray scale bitmap). The region of interest was marked from an upper 3mm region beginning 0.8mm proximal to the most proximal central epiphysis of the femur. For cortical bone analysis of femur (2D analysis), a 0.5mm region beginning 4.5mm proximal to the most proximal central epiphysis of the femur. 3D images were obtained from contoured 2D images by methods based on distance transformation of the grey scale original images (CT vox, Ver. 3.0.0). 3D and 2D analysis were performed using software CT Analyser (Ver. 1.15.4.0). All images presented are representative of the respective groups.

For histological analysis, the femurs were dissected and conducted in 4% PFA for 2 days. The they were placed to the 15% tetrasodium EDTA for 2 weeks so as to complete the procedure of decalcification. Moreover, slices of bone tissue were sectioned at 8 μm thickness along the coronal plate from anterior to posterior. The decalcified femur slices were stained with HE and masson. Respectively, immunohistochemical analysis was performed with heat-induced antigen retrieval in sodium-citrate buffer (Dako). Primary antibody about TRAP at 1:200. Biotinylated secondary antibody was used with the EnVision + system HRP kit (Dako) and nuclei were counterstained with hematoxylin.

### Statistical analysis

All data were representative of at least three experiments, which were analyzed and expressed as means ± standard deviation (SD). One-way ANOVA followed by Student-Newman-Keuls post hoc tests was used to determine the significance of difference between results. *p<0.05 and **p<0.01 were regarded as significant difference.
